# Theoretical Investigation of Halogen-Oxygen Bonding and Its Implications in Halogen Chemistry and Reactivity

**DOI:** 10.1155/2007/46393

**Published:** 2007-05-22

**Authors:** Agnie Mylona Kosmas

**Affiliations:** Division of Physical Chemistry, Department of Chemistry, University of Ioannina, 45110 Ioannina, Greece

## Abstract

Trends in the properties of normal valent and multivalent halogen-oxygen bonding are examined for the isomers of the halogen polyoxide families of the types (YXO2) and (YXO3), Y = Cl, Br, I, H, CH_3_, X = Cl, Br, I. A qualitative model is formulated on the relationship between the X−O bond distance variations, the ionic character of the bonding, and the degree of halogen valence. The relative stability and enthalpy of formation of each species are also suggested to correlate with the ionic nature of the X−O bonding and the electrostatic character of the Y, YO fragments. In the model presented, halogen hypervalence is interpreted to be the result of partial p → d promotion of lone-pair valence electrons followed by the formation of two, four, or six additional pd hybrid bonds around the halogen atom.

## 1. INTRODUCTION

High-level quantum mechanical electronic structure methodologies have known a spectacular development over the past two decades [1, 2], improving dramatically the accuracy of molecular geometry and energy calculations. For instance, it is currently possible to predict heat of formation values and reaction enthalpies to within 0.5 kcal mol^−1^, depending on the size of the system and the degree to which the molecule is well described by a single reference function. An area that has largely profited from this progress is the class of “highly correlated” molecules, that is, species possessing relatively low lying excited electronic states that affect the description of the ground or reference state through coupling from the correlation operator. A well-known set of highly correlated compounds are those that contain different electronegative elements bonded together as several species of atmospheric significance do, which contain halogen-oxygen bonds.

In stratospheric chemistry, halogen peroxides and their isomers are formed as intermediate complexes in the coupling of halogen monoxides

(1) $$\begin{array}{c} \text{RCl}\overset{\text{h}v}{\to }{\text{R}}^{•}+{\text{Cl}}^{•},\\ {\text{Cl}}^{•}+{\text{O}}_{3}\to {\text{ClO}}^{•}+{\text{O}}_{2},\\ {\text{ClO}}^{•}+{\text{ClO}}^{•}\to \text{ClOOCl}\to {\text{Cl}}^{•}+{\text{ClOO}}^{•},\\ {\text{ClO}}^{•}+{\text{ClO}}^{•}\to \text{ClOClO}\to {\text{Cl}}^{•}+{\text{OClO}}^{•},\\ {\text{ClO}}^{•}+{\text{BrO}}^{•}\to \text{BrOOCl}\to {\text{Br}}^{•}+{\text{ClOO}}^{•}. \end{array}$$

The halogen atoms thus released reattack new ozone molecules leading to a catalytic ozone depletion cycle. Systematic experimental studies have observed IR absorption spectra for several simple and cross halogen peroxides and polyoxides while some members have been isolated at 220° K which is the temperature prevailing in the stratospheric layer. The halogen monoxide radicals also interact with the hydroxyl HO^•^, methyl CH_3_^•^, hydroperoxy HO_2_^•^, and methylperoxy CH_3_O_2_^•^ radicals species abundant in the lower and upper atmosphere and give interesting reactions that participate in tropospheric ozone depletion cycles and in various hydrocarbon oxidation processes in atmospheric and combustion chemistry

(2) $$\begin{array}{c} {\text{HO}}^{•}+{\text{ClO}}^{•}\to \text{HOOCl}\to {\text{HO}}_{2}^{•}+{\text{Cl}}^{•}\to \text{HCl}+{\text{O}}_{2},\\ {\text{CH}}_{3}{\text{O}}_{2}^{•}+{\text{ClO}}^{•}\to {\text{CH}}_{3}\text{OOOCl}\to {\text{CH}}_{3}{\text{O}}^{•}+{\text{ClOO}}^{•}\to {\text{CH}}_{3}\text{OCl}+{\text{O}}_{2}. \end{array}$$

Due to their importance [3–9], the study of halogen polyoxides, (YXO2) and (YXO3), Y = Cl, Br, I, H, CH_3_, X = Cl, Br, I, has known intense theoretical and experimental investigations and has become one of the major chapters in halogen physical inorganic chemistry [10, 11].

The purpose of the present study is to examine the significant differences in the properties of normal valent and multivalent halogen-oxygen bonding in a series of chlorine, bromine and iodine containing polyoxides. This examination allows us to formulate a qualitative correlation model that links the structural variations and the energy stabilization tendencies among the various members with the degree of halogen valence, the ionicity of X–O bonding, and the electrostatic character of the Y, YO fragments.

## 2. COMPUTATIONAL DETAILS

As noted in the introduction, the quantum mechanical characterization of halogen polyoxides has been a subject of continuous and intense investigations in the recent literature. In the present work, the (YXO2) and (YXO3) isomers have been systematically optimized, all species at the same level of theory to allow a proper comparison of various molecular properties. More than fifty structures have been investigated in total. The MP2 method in combination with the 6-311G(d,p) basis set [12] has been used for all members of the Cl and Br families. For the treatment of I in the iodine polyoxides, the LANL2DZ basis set [13] has been employed, augmented with additional two-d and one-f polarization functions taken from the Stutgart-Bonn quantum chemistry package [14]. The computations have been carried out using the Gaussian 98 series of programs [15].

The calculated equilibrium geometries have been found consistent with the literature results wherever available [16–54], that have been obtained by employing either higher ab initio methodologies or density functional theory techniques combined with very large basis sets, depending on the computational resources available. For instance, Guha and Francisco have carried out the investigation of the (YBrO2), Y = H, Cl, Br, and (YBrO3), Y = H, CH_3_, isomers using the B3LYP functional in combination with the large 6-311++G(3df,3pd) basis set and the QCISD/6-31D(d) methodology [22, 34, 42]. In our laboratory, the members of the (CH_3_XO2) series, X = Cl, Br, I, have also been investigated at various levels of theory [40, 44, 48].

Regarding the energy computations, higher than MP2 theory calculations are required to make a reliable study of the energetics of all these systems. Opposite to the equilibrium geometry optimizations, accurate energy and relative stability computations for these adducts have been found to be very demanding and are very frequently quite sensitive to the theoretical method applied with severe discrepancies from one method to another. Such methodologies are beyond the capability of the present investigation, taking into account the large number of systems examined. However, the good agreement of the present optimizations, with various higher level structural calculations reported, allows us to use energy results previously published to analyze the dependence of the thermodynamic stability on the properties of X–O bonding. Thus, we discuss the relative energy and heat of formation tendencies and the correlation with the halogen valence and the Y, YO electronegativities, on the basis of collected literature results that have been obtained with the use of three different high level methodologies, namely, G2MP2, CCSD(T), and QCISD(T).

Harmonic vibrational frequencies have also been calculated along with the structural optimizations. They verify that all isomeric structures investigated represent energy minima on the corresponding potential energy surfaces. As an example, Table 1 summarizes the calculated harmonic vibrational frequencies for the isomers of the (ClBrO2) and (IClO3) families.

## 3. RESULTS AND DISCUSSION

Selected structural results for the (YClOn), (YBrOn), and (YIOn), Y = Cl, Br, I, H, CH_3_, *n* = 2, 3 isomers, calculated as described above, are collected in Tables 2–4. Depending on the position of the oxygen atom within the molecule, two kinds of X–O bonds (X = halogen atom) are distinguished, that is, with the O atom either located at a terminal position or within the molecule bound to another atom (bridged oxygen). When the X–O bond involves a terminal oxygen atom, it presents multiple bond properties and the corresponding halogen atom is characterized as hypervalent displaying more than 8 electrons in the valence shell. The interesting feature of the bonds between multivalent halogen atoms and terminal oxygen atoms is the severe tightening of the bond distance that does not correlate with the bond strength. Multiple X–O bonds exhibit a strikingly smaller equilibrium distance compared to the normal valent X–O length, a difference much more pronounced in the halogen case than, for instance, the difference in bond length between single and multiple carbon bonds.

### 3.1. Trends in the structural parameters of (YClO2), (YBrO2), and (YIO2) families, Y = Cl, Br, I, H, CH_3_

Three types of isomeric structures have been determined in this series, the peroxide normal valent YOOX form, the YOXO structure which contains both bridged and terminal oxygen-halogen bonds, and the fully hypervalent structure YXO_2_. The equilibrium X–O distances calculated present a wide range of values from the normal valent peroxide to the other isomers. For example, the Cl–O distance decreases from 1.756 Å in ClOOCl to 1.469 Å in ClClO_2_, a striking shrinkage of ∼0.3 Å. The ClOClO isomer presents three different types of Cl–O bonding. The bond that involves bridged Cl and O atoms is the longest, 1.909 Å, becoming 1.716 Å for the terminal Cl-bridged O bond and 1.512 Å between bridged Cl and terminal O, a net decrease of 0.4 Å. For comparison reasons, we may note that the Cl–O distance in free ClO radical has been calculated to be 1.576 Å at the same theory level. Similar large changes take place in all other halogen families with fully hypervalent halogen-terminal oxygen bond distance considerably tighter than the normal valent bonding. In the (YBrO2) family, for instance, the Br–O equilibrium length varies from 1.905 Å to 1.597 Å, again a range of the order of ∼0.3 Å.

### 3.2. Trends in the structural parameters of (YXO3) species

**Figure 1:**
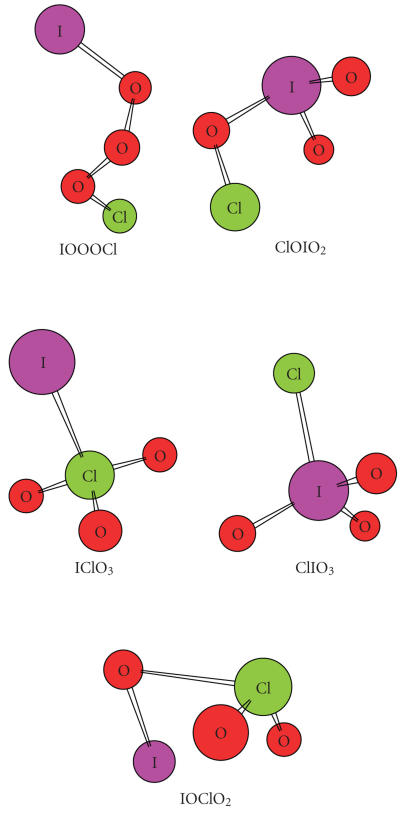
The optimized structures of (IClO3) isomers.

For Y = halogen atom, three types of isomers are obtained, the peroxide form, YOOOX, the fully hypervalent, YXO_3_, and the mixed one, YOXO_2_. As an example, Figure 1 displays the optimized structures of (IClO3) family. When Y = H, CH_3_, a fourth isomeric structure has been determined, that is, the HOOXO, CH_3_OOXO geometries. In all (YXO3), the largest X–O distances are found for the bonds in which both halogen and oxygen atoms are bridged while the shortest X–O bond lengths have been calculated for the fully hypervalent structures YXO_3_. The Cl–O bond distance in ClClO_3_, 1.418 Å, is the shortest ever calculated for a Cl–O bond while the bridged O-bridged Cl in ClOClO_2_, 2.032 Å, is the largest calculated for bound species. Similar large changes take place in the other halogen families and the same trends observed in (YXO2) are observed in the (YXO3). However, the deviations between largest and shortest bond lengths are even more emphasized in the (YXO3) than in the (YXO2) series.

### 3.3. Trends in relative energetics and thermodynamic stability

Relative energy values, displayed with respect to the electronic energies of the normal valent peroxide compounds, are summarized in Tables 5-6. They are mainly based on literature results calculated with the G2MP2 method but CCSD(T) and QCISD(T) reported values are also discussed when discrepancies with the G2MP2 values are encountered.

The general feature emerging from the inspection of the relative energy results is the absence of evidence for any correlation between the magnitude of the X–O bond length and the stabilization of the corresponding isomer. In fact, the fully hypervalent structures of the type YXO_3_ that contain the shortest X–O distances and the highest degree of halogen multivalence are the most unstable. On the other hand, the stabilization tendencies show an interesting dependence on the electronegativity of the Y, YO partners, a factor that must be examined in combination with the halogen partial atomic charge distributions. For example, the following Mulliken atomic charge distributions have been determined:

$$\begin{array}{c} \begin{array}{cc} \text{Cl}-\text{O}-\text{O}-\text{Cl,}-0.12/+0.12 & \text{Cl}-\text{Cl}-{\text{O}}_{2},-0.12/+1.17/-0.48\times 2 \end{array}\\ \text{H}-\text{Br}-{\text{O}}_{2}+0.05/+1.21/-0.63\times 2. \end{array}$$

These examples readily demonstrate that multivalent halogens exhibit considerable positive partial charge distributions increasing from hypervalent Cl to hypervalent I and indicating that the corresponding X–O bonds are highly ionic. A direct consequence is the increase in stabilization achieved when the strongly electropositively charged hypervalent halogen combines with an electronegative Y, YO partner. Several examples may be considered that demonstrate this correlation. For instance, the ClBrO_2_ structure is more stable than BrClO_2_ since the more electropositively charged hypervalent Br is connected to the more electronegative normal valent Cl. Another example is the YOClO_2_, YOBrO_2_, Y = H, CH_3_ compounds where the hypervalent, positively charged Cl, Br atoms connect to the highly electronegative YO fragments. These complexes compete in stability with the peroxide normal valent forms YOOOCl, YOOOBr, and their classification is very sensitive to the method applied. At the CCSD(T)/6-311G(d,p), QCISD(T)/6-311G(d,p) levels of theory [42, 43], they are placed only ∼1-2 kcal/mol above the normal valent peroxide structures, while at the G2MP2 level they are found to be even more stable by ∼2-3 kcal/mol [19, 34, 43]. The corresponding I containing isomer, the iodic acid HOIO_2_, presents particular stability and it is far more stable than the normal valent peroxide isomer HOOOI, as we will see in the next paragraph. On the other hand, the high instability of the fully hypervalent configurations HClO_2_, HBrO_2_, CH_3_ClO_2_, CH_3_BrO_2_, HClO_3_, HBrO_3_, CH_3_ClO_3_, CH_3_BrO_3_ may be readily explained when it is realized that the electropositively charged hypervalent halogen is necessarily connected to the electropositive H, CH_3_ partners. We suggest that the correlation of the ionic character of X–O bonding and the electrostatic character of the Y, YO fragments is a major factor affecting the stabilization of the corresponding polyoxide, that has not been given so far the appropriate attention required.

A related parameter that also influences the relative stability order appears to be the degree of valence on the halogen. The isomers ClOClO, BrOBrO are less stable than ClClO_2_, BrBrO_2_ despite having the hypervalent halogen also connected to an electronegative fragment such as ClO and BrO. The difference must most probably rely on the lower degree of halogen valence in the former compounds, which evidently plays also an important role in the stabilization process and becomes another significant factor affecting the stability of the compound. It has been suggested [11] that the multivalent configuration on the halogen is achieved when one, two, or three lone-pair valence electrons acquire a considerable amount of d character and form two, four, or six additional pd hybrid halogen bonds. The “energetic cost” of the first partial p→d promotion in the YOXO species is obviously only partly recovered by the X–O double bond. Once however, some pd hybridization has taken place, the energy level decreases and the mixing of the second lone-pair electrons is suggested [11] to occur much easier. This analysis [11] indicates that hypervalent halogen species with more than five bonds, such as the YXO_3_ species which contain seven bonds on the valence shell, will require the participation of the third halogen valence lone-pair electrons, that is, the s valence electrons [11] having already acquired some p character. Such a hybridization is obviously very costly and the YXO_3_ species are quite unstable thermodynamically as shown from the calculated results. We may conclude by saying that there appears to be an optimum degree of valence for stabilization which is five bonds around the halogen atom.

### 3.4. Particular features of the iodine oxides

The tendencies described above are most convincingly demonstrated in the hypervalent I series. The lower electronegativity of I compared to Cl, Br allows hypervalent iodine to carry a much larger positive charge distribution and enhances remarkably the ionic nature of I–O bonding. We have for example the following partial charge distributions:

$$\begin{array}{c} \text{H}-\text{O}-\text{O}-\text{I}\\ +0.44/-0.38/-0.39/+0.33\\ \text{H}-\text{O}-\text{I}-\text{O}\\ +0.47/-0.91/+1.37/-0.93\\ \text{H}-\text{I}-{\text{O}}_{2}\\ +0.12/+2.11/-0.99\times 2. \end{array}$$

As a result, the oxides containing hypervalent iodine are usually more stable than the normal valent isomers. Thus, in contrast to the YOClO, YOBrO species discussed above, YOIO isomers are more stable than the normal valent peroxide compounds YOOI, despite the lower degree of valence involved. In fact, the hypervalent I compounds are usually very stable thermodynamically and one of them, iodic acid HOIO_2_, a white powdered solid, is even stable at room temperature. Also methyl iodate CH_3_OIO_2_, because of its thermodynamic stability, is found to operate as a reservoir species for iodine in the marine boundary layer [52]. The only exceptions are the fully hypervalent HIO_2_, HIO_3_, CH_3_IO_2_, CH_3_IO_3_ isomers where the hypervalent I may only form a weak bond with the electropositive H, CH_3_ fragments producing also unstable adducts like the Cl, Br analogues.

### 3.5. Heat of formation values

The most appropriate measure of the actual thermodynamic stability of chemical species is the heat of formation values, ΔH_*f*_, which allow a direct comparison among various compounds. Several computed values in the present laboratory as well as various literature results are summarized in Table 7. For comparison the maternal compound, HOOH, is also included. The table shows that low heat of formation values are calculated for the normal-valent cross hydrogen-halogen peroxides of the type HOOX. Among the multivalent species low heat of formation values are computed for the acidic derivatives HOXO, HOXO_2_, and the methyl analogs CH_3_OXO_2_, X = Cl, Br, I, which may be considered relatively stable at room temperature. The polyoxides that involve two halogen atoms are usually unstable and present very short lifetimes, having been detected only spectroscopically as bound short-lived intermediates in the kinetic investigation of various atmospheric processes or in matrix isolation studies. As a rule the iodine containing species are the most stable halogen polyoxides.

## 4. SUMMARY

The halogen-oxygen bonding properties in the (YXO2) and (YXO3) polyoxides, Y = Cl, Br, I, H, CH_3_ and X = Cl, Br, I, have been examined. The analysis shows that multivalent bonding presents a strong ionic nature and affects the structural characteristics and the thermodynamic stabilization trends. The X–O bond distance decreases considerably in the fully hypervalent compounds but its variations do not correlate with energy stability order. From the analysis presented, the thermodynamic stability and the relative stability order of the various isomers are suggested to result from the combination of three factors: (a) the electrostatic nature of the Y, YO fragments, (b) the electronegativity of the halogen, and (c) the degree of halogen valence in the formation of the hypervalent bonds.

## Figures and Tables

**Table 1:** Harmonic vibrational frequencies (cm^−1^) for the isomers of (ClBrO2), (ClIO3) families.

ClOOBr	776, 676, 595, 431, 296, 103
ClBrO_2_	832, 331, 297, 211, 876, 168
BrClO_2_	1182, 528, 407, 192, 1335, 212
ClOBrO	795, 575, 348, 224, 174, 90
BrOClO	1162, 574, 445, 338, 215, 91
	
ClOOOI	853, 746, 651, 580, 55 3, 435, 212, 144, 52
ClOIO_2_	965, 952, 774, 465, 332, 290, 238, 136, 45
IOClO_2_	1231, 995, 675, 494, 443, 359, 221, 175, 101
ClIO_3_	920, 392, 295, 981, 308, 195
IClO_3_	1075, 624, 218, 1290, 527, 232

**Table 2:** Bridged and terminal Cl–O bond distances (Å) in (YClO2) and (YClO3) isomeric structures.

Species	ClOOCl	ClOClO	ClClO_2_	
Cl–O bridged	1.756	1.909		
Cl–O terminal		1.512	1.469	
Terminal Cl–O		1.713		

Species	BrOOCl	BrOClO	BrClO_2_	
Cl–O bridged	1.703	1.735		
Cl–O terminal		1.489	1.430	

Species	IOOCl	IOClO	IClO_2_	
Cl–O bridged	1.716	1.749		
Cl–O terminal		1.492	1.450	

Species	HOOCl	HOClO	HClO_2_	
Cl–O bridged	1.739	1.754		
Cl–O terminal		1.513	1.488	

Species	CH_3_OOCl	CH_3_OClO	CH_3_ClO_2_	
Cl–O bridged	1.761	1.763		
Cl–O terminal		1.520	1.489	

Species	ClOOOCl	ClOClO_2_	ClClO_3_	
Cl–O bridged	1.704	2.032		
Cl–O terminal		1.464	1.418	
Terminal Cl–O		1.632		

Species	BrOOOCl	BrOClO_2_	BrClO_3_	
Cl–O brigdged	1.725	2.045		
Cl–O terminal		1.440	1.424	

Species	IOOOCl	IOClO_2_	IClO_3_	
Cl–O bridged	1.707	2.097		
Cl–O terminal		1.460	1.426	

Species	HOOOCl	HOOClO	HOClO_2_	HClO_3_
Cl–O bridged	1.750	1.874	1.754	
Cl–O terminal		1.511	1.452	1.427

Species	CH_3_OOOCl	CH_3_OOClO	CH_3_OClO_2_	CH_3_ClO_3_
Cl–O bridged	1.742	1.864	1.786	
Cl–O terminal		1.510	1.473	1.461

**Table 3:** Bridged and terminal Br–O bond distances (Å) in (YBrO2) and (YBrO3) isomeric structures.

Species	BrOOBr	BrOBrO	BrBrO_2_	
Br–O bridged	1.878	1.897		
Br–O terminal		1.648	1.618	
Terminal Br–O		1.815		

Species	ClOOBr	ClOBrO	ClBrO_2_	
Br–O bridged	1.884	1.892		
Br–O terminal		1.659	1.613	

Species	IOOBr	IOBrO	IBrO_2_	
Br–O bridged	1.870	1.872		
Br–O terminal		1.634	1.597	

Species	HOOBr	HOBrO	HBrO_2_	
Br–O bridged	1.883	1.868		
Br–O terminal		1.676	1.645	

Species	CH_3_OOBr	CH_3_OBrO	CH_3_BrO_2_	
Br–O bridged	1.899	1.905		
Br–O terminal		1.666	1.647	

Species	ClOOOBr	ClOBrO_2_	ClBrO_3_	
Br–O bridged	1.887	2.084		
Br–O terminal		1.613	1.601	

Species	IOOOBr	IOBrO_2_	IBrO_3_	
Br–O bridged	1.859	2.167		
Br–O terminal		1.610	1.582	

Species	HOOOBr	HOOBrO	HOBrO_2_	HBrO_3_
Br–O bridged	1.867	1.919	1.844	
Br–O terminal		1.635	1.598	1.586

Species	CH_3_OOOBr	CH_3_OOBrO	CH_3_OBrO_2_	CH_3_BrO_3_
Br–O bridged	1.898	1.977	1.882	
Br–O terminal		1.653	1.620	1.615

**Table 4:** Bridged and terminal I–O bond distances (Å) in (YIO2) and (YIO3) isomeric structures.

Species	IOOI	IOIO	IIO_2_	
I–O bridged	2.065	2.066		
I–O terminal		1.820	1.790	
Terminal I–O		2.046		

Species	ClOOI	ClOIO	ClIO_2_	
I–O bridged	2.060	2.064		
I–O terminal		1.818	1.783	

Species	BrOOI	BrOIO	BrIO_2_	
I–O bridged	2.064	2.065		
I–O terminal		1.819	1.786	

Species	HOOI	HOIO	HIO_2_	
I–O bridged	2.058	1.992		
I–O terminal		1.815	1.784	

Species	CH_3_OOI	CH_3_OIO	CH_3_IO_2_	
I–O bridged	2.005	1.948		
I–O terminal		1.777	1.749	

Species	ClOOOI	ClOIO_2_	ClIO_3_	
I–O bridged	2.018	2.001		
I–O terminal		1.744	1.733	

Species	BrOOOI	BrOIO_2_	BrIO_3_	
I–O bridged	2.019	1.980		
I–O terminal		1.744	1.735	

Species	HOOOI	HOOIO	HOIO_2_	HIO_3_
I–O bridged	2.012	1.982	1.919	
I–O terminal		1.835	1.795	1.794

Species	CH_3_OOOI	CH_3_OOIO	CH_3_OIO_2_	CH_3_IO_3_
I–O bridged	2.003	1.992	1.994	
I–O terminal		1.786	1.762	1.738

**Table 5:** Relative stabilities of (YXO2) isomers (kcal mol^−1^) compared to the normal valent peroxide species YOOX in each family^(a)^.

Species	ClOClO	BrOClO	IOClO	HOClO	CH_3_OClO
ΔE	14.5^(a)^	10.5^(c)^	12.4^(e)^	8.3^(f)^	8.4^(g)^
	13.3^(b)^	5.2^(d)^			

Species	BrOBrO	ClOBrO	IOBrO	HOBrO	CH_3_OBrO
ΔE	11.0^(h)^	13.1^(c)^	3.8^(e)^	2.9^(d)^	6.3^(i)^
	8.7^(d)^	8.6^(d)^			

Species	ClOIO	BrOIO	IOIO	HOIO	CH_3_OIO
ΔE	−5.0^(e)^	−6.4^(e)^	−8.2^(e)^	−13.2^(k)^	−0.2^(l)^
			−7.9^(j)^		

Species	ClClO_2_	BrClO_2_	IClO_2_	HClO_2_	CH_3_ClO_2_
ΔE	5.1^(a)^	6.8^(c)^	22.6^(e)^	49.7^(f)^	30.8^(g)^
	3.4^(b)^	7.8^(d)^			

Species	BrBrO_2_	ClBrO_2_	IBrO_2_	HBrO_2_	CH_3_BrO_2_
ΔE	8.2^(h)^	3.4^(c)^	9.3^(e)^	52.6^(d)^	33.4^(i)^
	6.8^(d)^	2.4^(d)^			

Species	IIO_2_	ClIO_2_	BrIO_2_	HIO_2_	CH_3_IO_2_
ΔE	−14.6^(e)^	−32.3^(e)^	−23.6^(e)^	59.5^(k)^	18.5^(l)^
	−12.9^(j)^				

^(a)^Reference [37], ^(b)^reference [16], ^(c)^reference [36], ^(d)^reference [22], ^(e)^reference [39], ^(f)^reference [17], ^(g)^reference [44], ^(h)^reference [29], ^(i)^reference [40], ^(j)^reference [25], ^(k)^reference [45], ^(l)^reference [48].

**Table 6:** Relative stabilities of (YXO3) isomers in kcal mol^−1^ compared to the normal valent peroxide species YOOOX in each family.

Species	ClOClO_2_	BrOClO_2_	IOClO_2_	HOOClO	HOClO_2_	CH_3_OOClO	CH_3_OClO_2_
ΔE	−2.2^(a)^	−3.0^(c)^	−5.7^(a)^	21.1^(d)^	−2.8^(d)^	12.4^(e)^	−7.9, 4.2^(f)^
	−2.1^(b)^						

Species	ClOBrO_2_	IOBrO_2_	HOOBrO	HOBrO_2_	CH_3_OOBrO	CH_3_OBrO_2_	
ΔE	−0.5^(c)^	−14.1^(a)^	14.5^(g)^	−5.7^(g)^	12.3^(h)^	−5.6^(h)^, 1.4^(i)^	

Species	ClOIO_2_	BrOIO_2_	HOOIO	HOIO_2_	CH_3_OOIO	CH_3_OIO_2_	
ΔE	−37.4^(a)^	−39.7^(a)^	−0.1^(j)^	−39.9^(j)^	−1.3^(k)^	−31.2^(k)^	

Species	ClClO_3_	BrClO_3_	IClO_3_	HClO_3_	CH_3_ClO_3_		
ΔE	10.6^(a)^	8.1^(c)^	23.4^(a)^	42.7^(d)^	47.2^(f)^		
	4.6^(b)^						

Species	ClBrO_3_	IBrO_3_	HBrO_3_	CH_3_BrO_3_			
ΔE	16.2^(c)^	10.5^(a)^	59.8^(g)^	37.2^(h)^			

Species	ClIO_3_	BrIO_3_	HIO_3_	CH_3_IO_3_			
ΔE	−35.5^(a)^	−27.3^(a)^	29.9^(j)^	13.3^(k)^			

^(a)^Reference [46], ^(b)^reference [23], ^(c)^reference [24], ^(d)^reference [19], ^(e)^reference [43], ^(f)^reference [43] at the G2MP2 and the CCSD(T), QCISD(T) levels, respectively, ^(g)^reference [27], ^(h)^reference [34], ^(i)^reference [42], ^(j)^reference [49], ^(k)^reference [53].

**Table 7:** Collected heat of formation Δ H*_f_* ^°^(kcal mol^−1^) reported values for several halogen polyoxides^(a)^.

(YXO2)	Δ H*_f_* ^°^	(YXO3)	Δ H*_f_* ^°^
HOOH	−31.0	BrOOOCl	49.8
ClClO_2_	33.8	BrOClO_2_	46.8
ClOClO	40.0	ClOBrO_2_	49.3
ClOOCl	32.6	HOOOCl	9.1
HOClO	11.9	HOOClO	25.3
HClO_2_	56.1	HOClO_2_	4.2
HOOCl	1.6	HClO_3_	46.1
HOOBr	8.6	BrClO_3_	57.9
HOBrO	13.2	ClBrO_3_	66.0
HBrO_2_	63.7	ClOOOCl	40.3
ClOOBr	38.9	ClOClO_2_	38.2
ClOBrO	48.3	ClClO_3_	44.9
BrOClO	49.4	HOOOBr	13.6
ClBrO_2_	41.9	HOBrO_2_	5.7
BrClO_2_	45.7	HOOBrO	26.2
BrOOBr	46.1	HBrO_3_	71.6
BrBrO_2_	52.9	BrOBrO_2_	56.4
BrOBrO	54.8	CH_3_OOOBr	16.2
HOOI	7.6	CH_3_OOBrO	28.5
HOIO	−5.3	CH_3_OBrO_2_	10.6
HIO_2_	26.5	CH_3_BrO_3_	53.4
IOOI	38.7	HOOOI	21.9
IOIO	30.8	HOOIO	21.6
IIO_2_	25.8	HOIO_2_	−17.8
		HIO_3_	51.0

^(a)^Reference [16–53].
